# Supplementary material for: An archaeal nucleoid-associated protein binds an essential motif in DNA replication origins
Source: Nat Commun. 2025 Jun 5;16:5230. doi: 10.1038/s41467-025-60618-3 (PMC12141716; doi:10.1038/s41467-025-60618-3)
Supplement: Supplementary file 1 — Supplementary Information [file 41467_2025_60618_MOESM1_ESM.pdf]

Supplementary Information

An archaeal nucleoid-associated protein binds an essential motif in DNA replication origins

Rajkumar Dhanaraju[1], Rachel Y. Samson[1,2,3,4], Xu Feng[1,5], Alessandro Costa[3,6], Giovanni Gonzalez-Gutierrez[1] and Stephen D. Bell[1,2,3,4*]

[1] Department of Molecular and Cellular Biochemistry, Indiana University, Simon Hall MSB1, 212 S Hawthorne Drive, Bloomington, IN 47405, USA

[2] Department of Biology, Indiana University, Simon Hall MSB1, 212 S Hawthorne Drive Bloomington, IN 47405, USA

[3] Sir William Dunn School of Pathology, South Parks Road, Oxford, OX1 3RE, UK

[4] Present address. Department of Microbiology, The Ohio State University, Aronoff Laboratory 218, 318 W 12th Ave, Columbus, OH, 43210, USA

[5] Present address. State Key Laboratory of Microbial Technology, Shandong University, Qingdao, 266237, China

[6] Present address. Macromolecular Machines Laboratory, The Francis Crick Institute, London, UK

*Corresponding author: Stephen D Bell (bell.2007@osu.edu)

Supplementary information contains

Supplementary Figure 1 – 7

Supplementary Tables 1 and 3

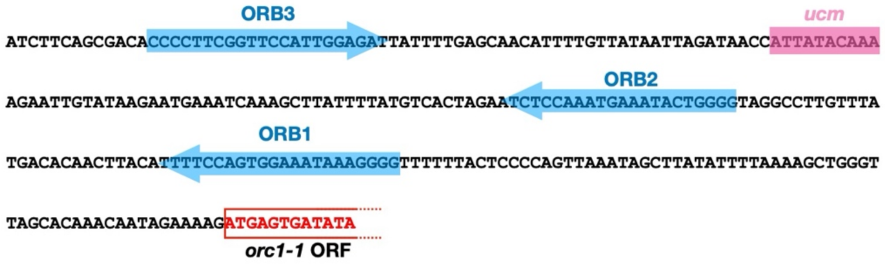

Supplementary Figure 1. *Sulfolobus islandicus oriC1* sequence.

Sequence of *Sulfolobus islandicus* REY15A *oriC1* with the ORB elements bound by Orc1-1 highlighted with blue arrows and the position of the *ucm* in magenta. The initial 12 nucleotides of the *orc1-1* open reading frame are highlighted in red.

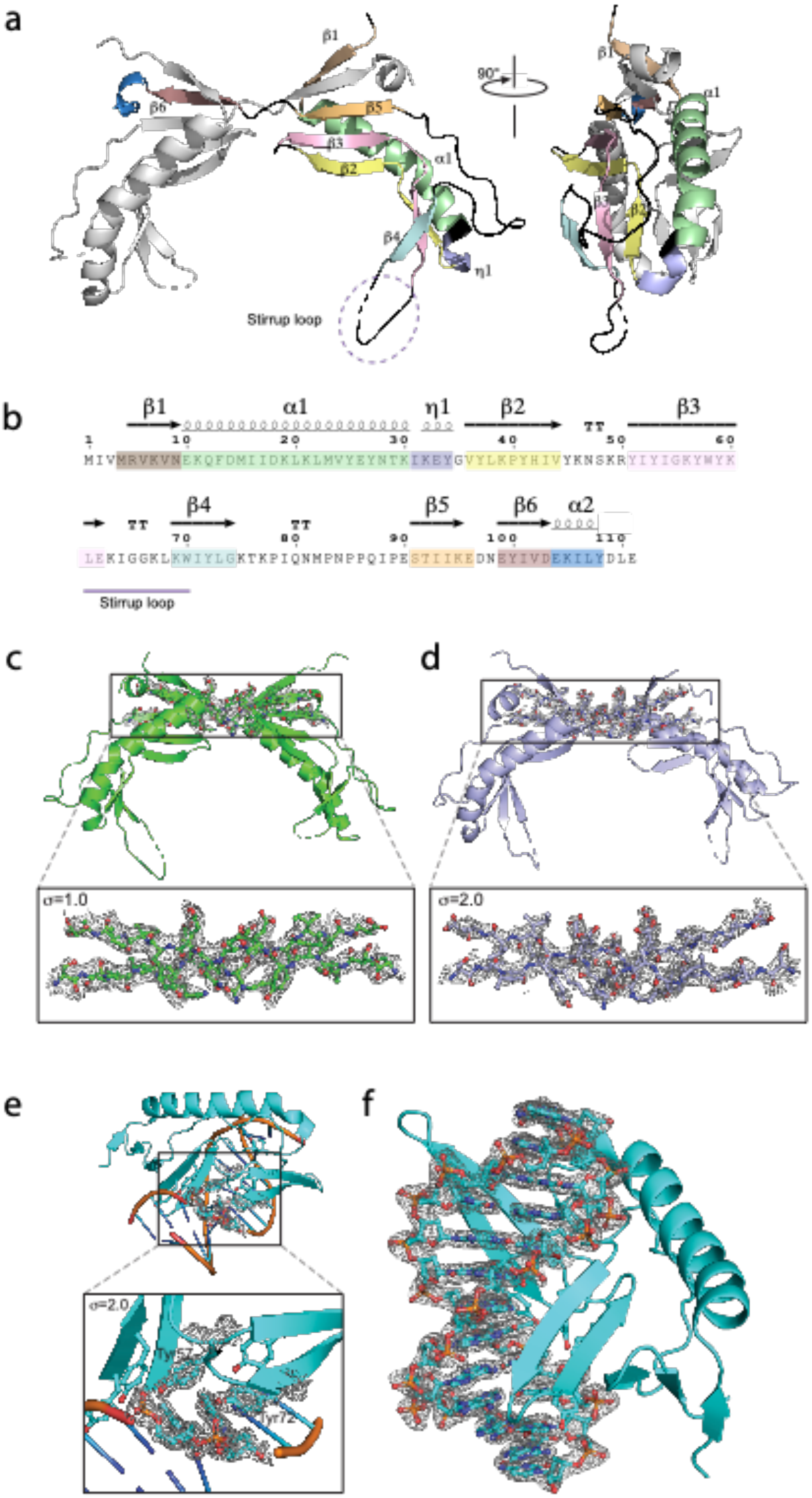

Supplementary Figure 2. Structure of UBP.

a. Secondary structure elements of one protomer are colored and labeled in agreement with the sequence in panel b

b. Secondary structure elements annotated using the ESPript 3.0 website, https://espript.ibcp.fr/ESPript/ESPript/).

c. X-ray crystal structure of the DNA-free UBP homodimer. (inset) Close-up of the C-terminal strand swap interface of the UBP homodimer, showing the electron density map as a gray mesh countered at σ=1.0. Tyr100 for both monomers are shown as reference.

d. X-ray crystal structure of the DNA-free UBP homodimer wide form. (inset) Close-up of the C-terminal strand swap interface of the UBP homodimer, showing the electron density map as a gray mesh countered at σ=2.0. Tyr100 for both monomers are shown as reference.

e. X-ray crystal structure of UBP bound to duplex oligonucleotides corresponding to the *ucm* sequence. (inset) Close-up of Tyr57 and Tyr72 interacting with phosphate groups of a single oligonucleotide as example of several Tyr – DNA interactions observed in the structure. The electron density map as a gray mesh is countered at σ=2.0.

f. X-ray crystal structure of UBP bound to duplex oligonucleotides corresponding to the *ucm* sequence (as sticks representation). The gray mesh corresponds to the composite omit map countered at σ=2.0.

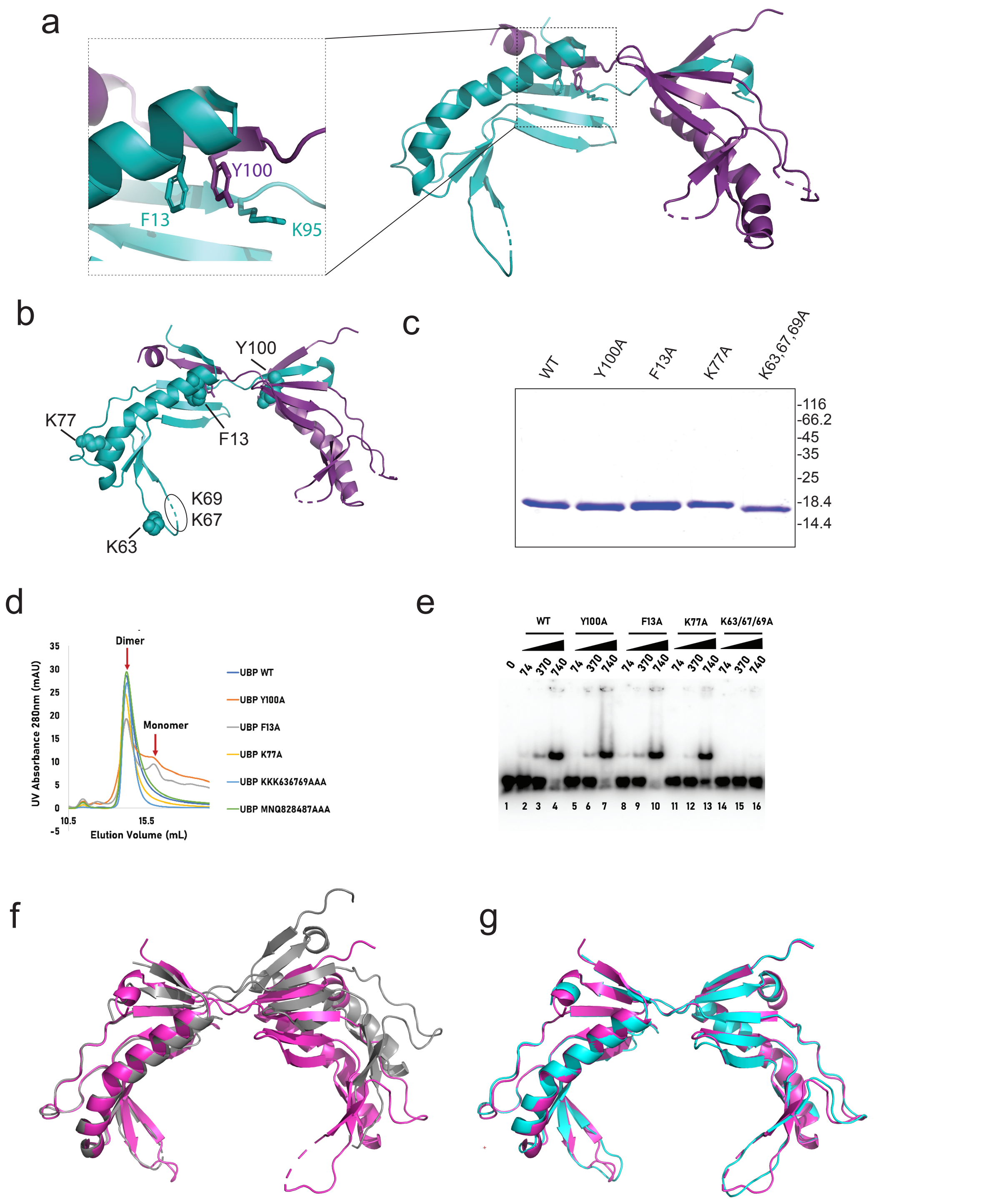

Supplementary Figure 3. Structure based mutagenesis of UBP

a. Expanded view of the dimerization interface between UBP protomers with key residues F13 and K95 from one protomer in teal and Y100 of the partner protein in purple.

b. Position of residues targeted for mutagenesis.

c. Coomassie-stained SDS_PAGE analysis of purified UBP proteins with the indicated mutations (WT – wild-type).

d. Size exclusion chromatography of wild-type and the indicated mutated forms of UBP. Positions of peaks corresponding to monomer and dimer forms of the protein are indicated.

e. EMSAs with the various mutated forms of UBP on a *ucm*-containing DNA duplex oligonucleotide.

f. Overlay of the structures of UBP dimers from the crystals with space group P21.

g. Overlay of the original structure described in Figure 3 and Supplementary Figure 3a (cyan) with the magenta dimer from the panel Supplementary Figure 3f.

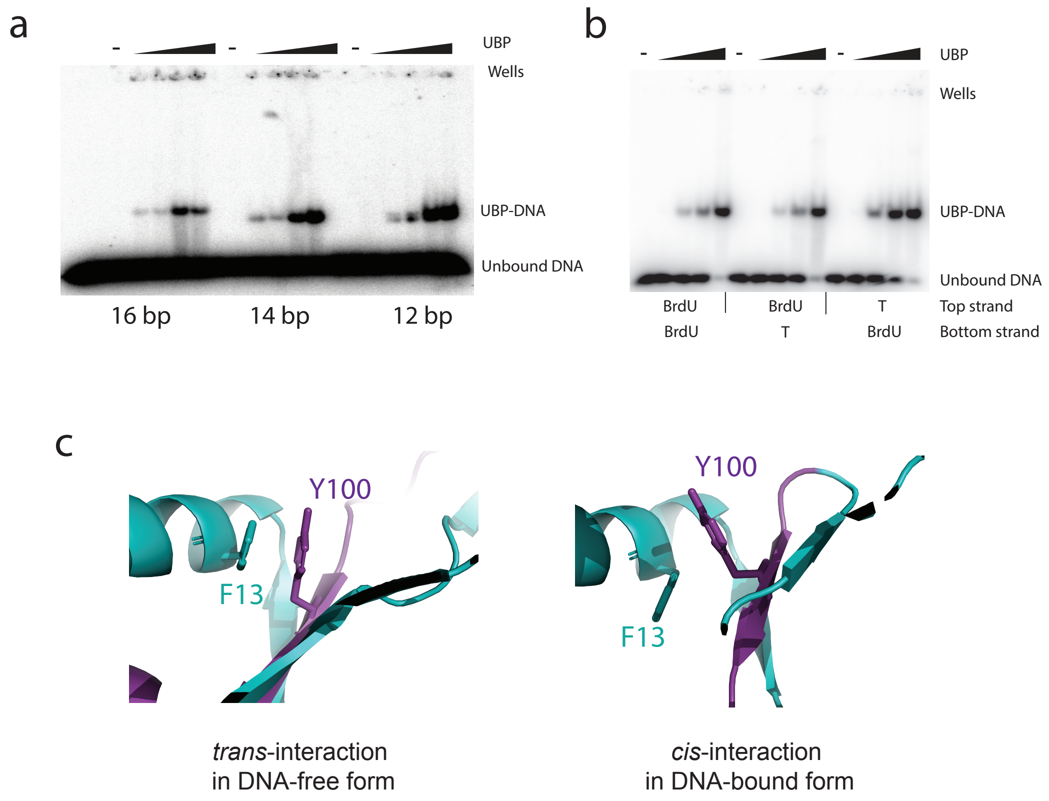

Supplementary Figure 4. UBP-DNA interactions

a. EMSAs with dsDNA probes centered on the *ucm* of total length 16, 14 and 12 bp. Reactions contained 0, 74, 370, 740 or 1480 nM UBP.

b. EMSAs with 12 bp dsDNA probes with BrdU substitutions for T on top (5' ABrdUTABrdUACAAAAG 3') and/or bottom (5' CBrdUTTTGTABrdUAAT 3') strands as indicated. Reactions contained 0, 74, 370, 740 or 1480 nM UBP.

c. Comparison on the switch between planar and T-shaped stacking interactions between F13 and Y 100 in the interactions inter-molecular interaction in the DNA-free dimer form and the intra-molecular interaction in the monomeric DNA-bound form.

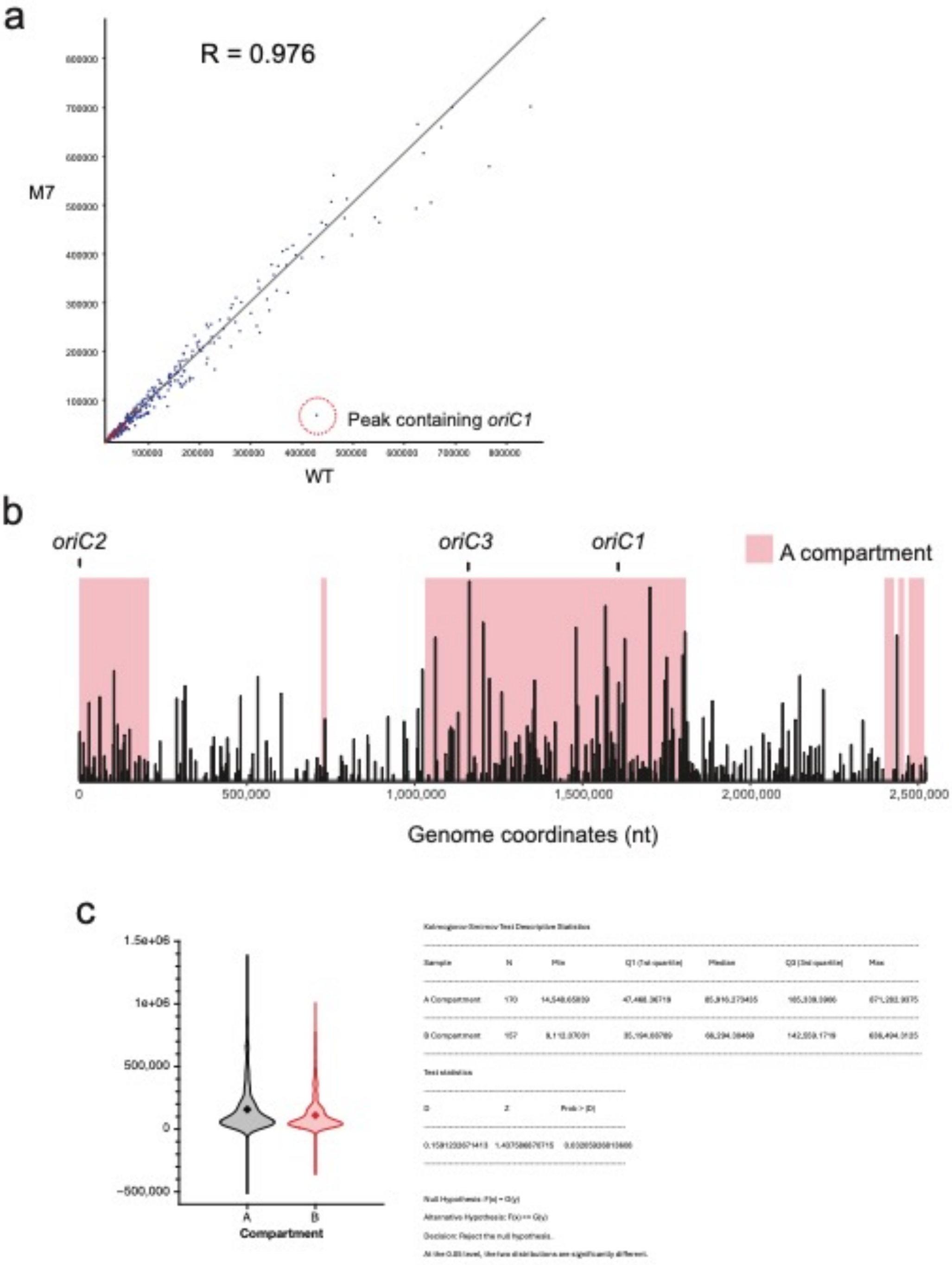

Supplementary Figure 5. ***in vivo*** **chromosome occupancy of UBP**

a. Comparison of ChIP-Seq peaks between the wild-type and M7 (*ucm* mutant) with the Pearson correlation value given. The peak corresponding to *oriC1* is highlighted.

b. ChIP-Seq profile in wild-type cells shown with the positions of the A-compartment highlighted in pink (compartment coordinates extracted from data presented in [25])

c. Violin plot and Kolmogorov-Smirnov test indicating a significant enrichment of UBP ChIP-Seq signal in the A compartment.

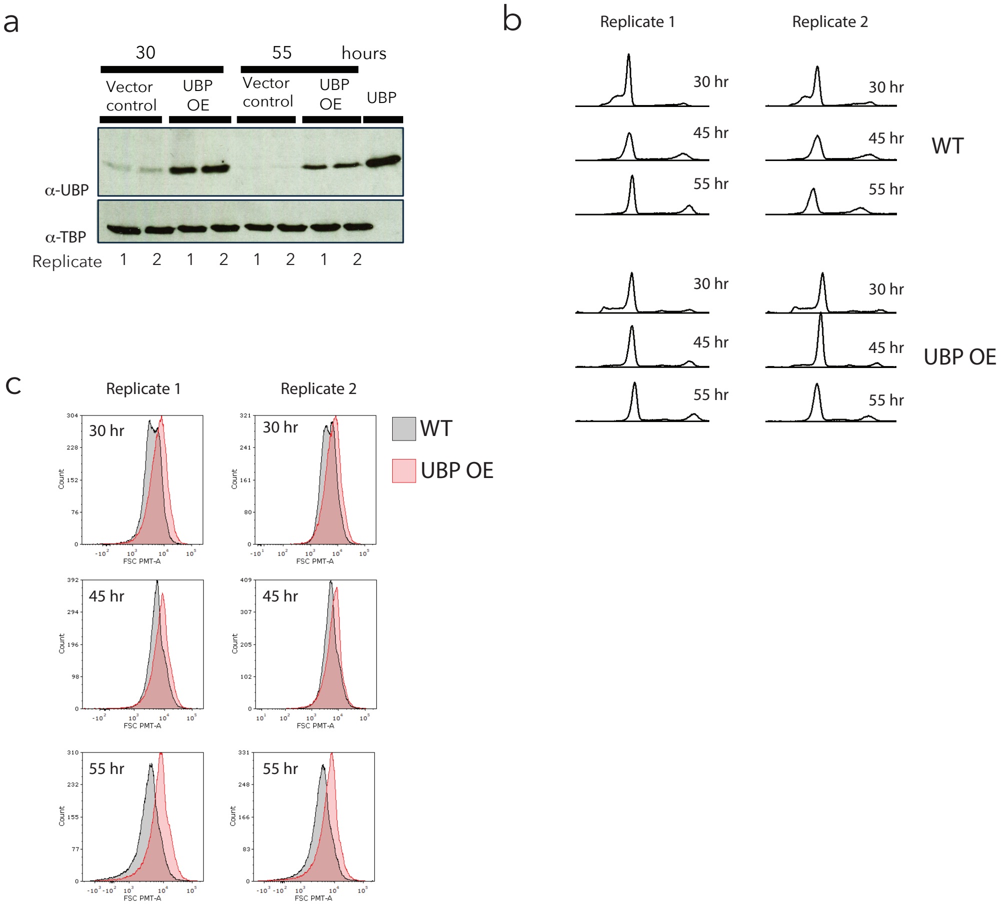

Supplementary Figure 6. UBP over-expression *in vivo*

a. Western blotting confirming the over-expression of UBP in both replicate cultures of the UBP-over-expression strain compared to the empty vector strains at 30 and 55 hour time points. Recombinant UBP is added as a positive control at the right-hand side of the gel. Western blotting for the general transcription factor TBP was used as a loading control (lower panel)

b. Flow cytometry profiles for the replicate cultures for both vector and UBP-over-expression strains at the indicated time points (replicate 1 is also shown in main Figure 6b).

c. Forward scatter profiles for the replicate cultures described in the above panel (Supplementary Figure 6b) at the indicted timepoints for cells with te empty vector (gray) or over-expressing UBP (pink).

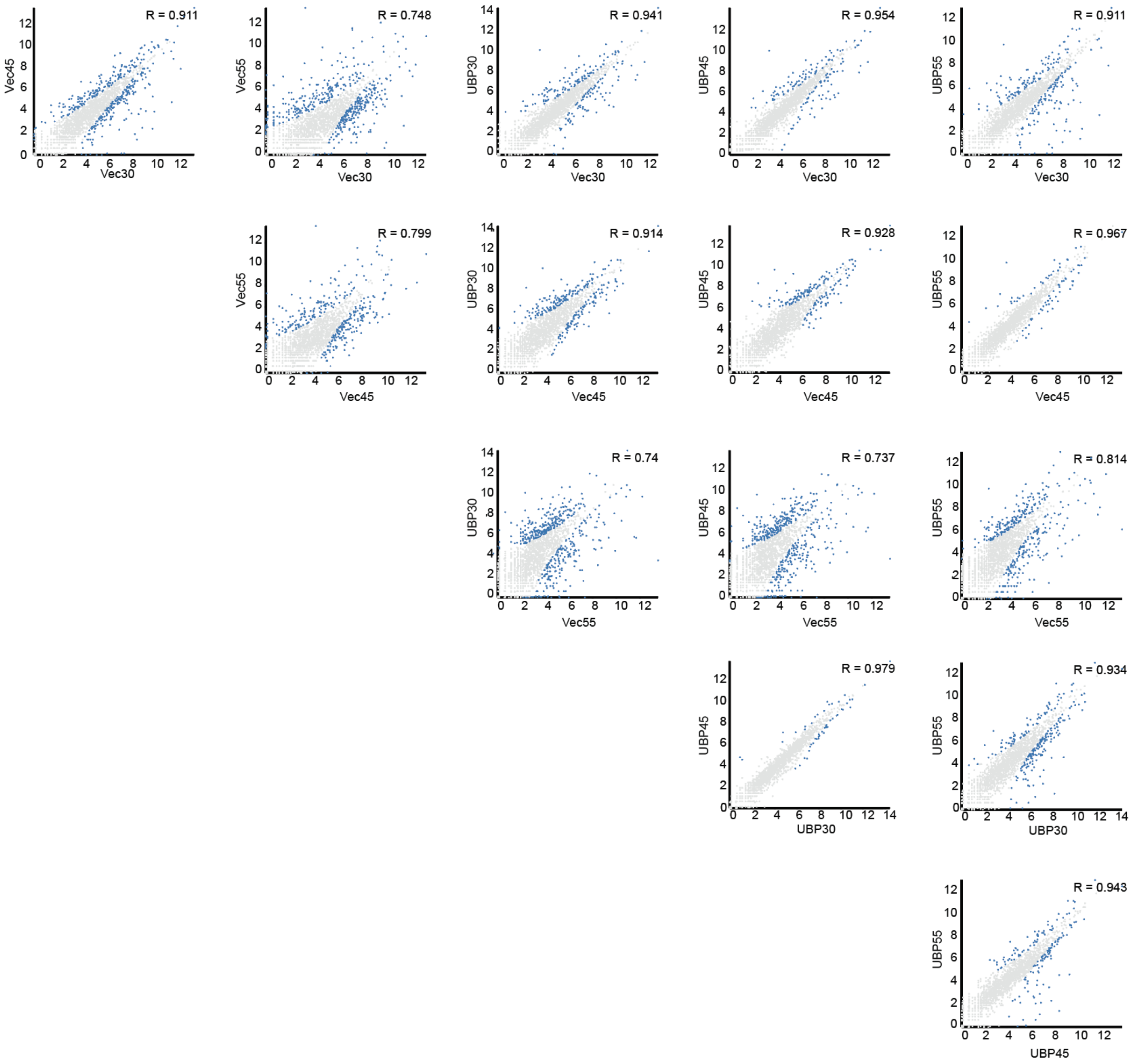

Supplementary Figure 7. Pairwise comparisons of transcriptome changes upon UBP over-expression

Scatter plots of log 2 values for gene expression in the indicated pairs of strains. Genes with differential expression (DESeq2 p<0.05) are indicated in blue.

Supplementary Table 1. List of oligonucleotides used in this study.

UCMwt-EMSAf	TGTTATAATTAGATAACCATTATACAAAAG AATTGTATAA
UCMwt-EMSAr	TTATACAATTCTTTTGTATAATGGTTATCTAATTATAACA
UCMm7-EMSAf	TGTTATAATTAGATAACCATGCGCACCCCTAATTGTATAA
UCMm7-EMSAr	TTATACAATTAGGGGTGCGCATGGTTATCTAATTATAACA
DNA probe used for Footprinting assay	ggccgcCAAGGCCTACCCCAGTATTTCATTTGGAGATTCTAGTGAC ATAAAATAAGCTTTGATTTCATTCTTATACAATTCTTTTGTATAAT GGTTATCTAATTATAACAAAATGTTGCTCAAAATAATCTCCAATGG AACCGAAGGGGTGTCGCTGAGGATCCCCCGGGCTGCAggaatt
UCM mutations (Transversions)	
M1	ACCCCTTCGG to CAAAAGGATT
M1Spf	aagGTTCATATCTTCAGCGACACCCCTTCGGTTCCATTGGAGA
M1Spr	agcTCTCCAATGGAACCGAAGGGGTGTCGCTGAAGATATGAAC
ucmDonor-f	tttgcatgcGTCAATCCTAAATGTCCCGTATGTG
M1Donor-SOEr	CAATGGAAaatccttttgGTCGCTGAAGATATGAAC
M1Donor-SOEf	CAGCGACcaaaaggattTTCCATTGGAGATTATTTTG
ucmDonor-r	gtttctcgagCAAAAGCGTCTATCTCATCCAAAAC
M2	TTCCATTGGA to GGAACGGTTC
M2Spf	aagGCGACACCCCTTCGGTTCCATTGGAGATTATTTTGAGCAA
M2Spr	agcTTGCTCAAAATAATCTCCAATGGAACCGAAGGGGTGTCGC
M2 Donor-SOEr	AAATAATCgaaccgttccCCGAAGGGGTGTCGCTGAAG
M2 Donor-SOEf	CCTTCGGggaacggttcGATTATTTTGAGCAACATTT
M3	GATTATTTTG to TCGGCGGGGT
M3Spf	aagCTTCGGTTCCATTGGAGATTATTTTGAGCAACATTTTGTT
M3Spr	agcAACAAAATGTTGCTCAAAATAATCTCCAATGGAACCGAAG
M3Donor-SOEr	TGTTGCTaccccgccgaTCCAATGGAACCGAAGGGGT
M3Donor-SOEf	CCATTGGAtcggcggggtAGCAACATTTTGTTATAATT
M4	AGCAACATTT to CTACCACGGG
M4Spf	aagTTGGAGATTATTTTGAGCAACATTTTGTTATAATTAGATA
M4Spr	agcTATCTAATTATAACAAAATGTTGCTCAAAATAATCTCCAA
M4Donor-SOEr	TTATAACAcccgtaatagCAAAATAATCTCCAATGGAA
M4Donor-SOEf	GATTATTTTGctattacgggTGTTATAATTAGATAACCAT
M5	TGTTATAATT to GTGGCGCCGG
M5Spf	the same to M4Spf
M5Spr	the same to M4Spr
M5Donor-SOEr	GGTTATCTccggcgccacAAATGTTGCTCAAAATAATCTCC
M5Donor-SOEf	CAACATTTgtggcgccggAGATAACCATTATACAAAAG
M6	AGATAACCAT to CTCGCCAACG
M6Spf	aagTATACAATTCTTTTGTATAATGGTTATCTAATTATAACAA
M6Spr	agcTTGTTATAATTAGATAACCATTATACAAAAGAATTGTATA
M6Donor-SOEr	TTTGTATAcgttggcgagAATTATAACAAAATGTTGCTC
M6Donor-SOEf	GTTATAATTctcgccaacgTATACAAAAGAATTGTATAAG
M7	TATACAAAAG to GCGCACCCCT
M7Spf	aagTTCTTATACAATTCTTTTGTATAATGGTTATCTAATTATA
M7Spr	agcTATAATTAGATAACCATTATACAAAAGAATTGTATAAGAA
M7Donor-SOEr	ATACAATTaggggtgcgcATGGTTATCTAATTATAACA
M7Donor-SOEf	GATAACCATgcgcacccctAATTGTATAAGAATGAAATC
M8	AATTGTATAA to CCGGTGCGCC
M8Spf	aagTTATACAAAAGAATTGTATAAGAATGAAATCAAAGCTTAT

M8Spr	agcATAAGCTTTGATTTCATTCTTATACAATTCTTTTGTATAA
M8Donor-SOEr	TTTCATTCggcgcaccggCTTTTGTATAATGGTTATC
M8Donor-SOEf	ACAAAAGccggtgcgccGAATGAAATCAAAGCTTATT
M9	GAATGAAATC to TCCGTCCCGA
M9Spf	The same as M8Spf
M9Spr	The same as M8Spr
M9Donor-SOEr	TAAGCTTTtcgggacggaTTATACAATTCTTTTGTAT
M9Donor-SOEf	TGTATAAtccgtcccgaAAAGCTTATTTTATGTCAC
M10	AAAGCTTATT to CCCTAGGCGG
M10Spf	aagTTTGGAGATTCTAGTGACATAAAATAAGCTTTGATTTCAT
M10Spr	agcATGAAATCAAAGCTTATTTTATGTCACTAGAATCTCCAAA
M10Donor-SOEr	GACATAAccgcctagggGATTTCATTCTTATACAATT
M10Donor-SOEf	TGAAATCccctaggcggTTATGTCACTAGAATCTCCA
M11	TTATGTCACT to GGCGTGACAG
M11Spf	aagAAGCTTATTTTATGTCACTAGAATCTCCAAATGAAATACT
M11Spr	agcAGTATTTCATTTGGAGATTCTAGTGACATAAAATAAGCTT
M11Donor-SOEr	GAGATTCTctgtcacgccAATAAGCTTTGATTTCATTC
M11Donor-SOEf	GCTTATTggcgtgacagAGAATCTCCAAATGAAATAC
M12	AGAATCTCCA to CTCCGAGAAC
M12Spf	The same as M11Spf
M12Spr	The same as M11Spr
M12Donor-SOEr	ATTTCATTgttctcggagAGTGACATAAAATAAGCTTT
M12Donor-SOEf	TGTCACTctccgagaacAATGAAATACTGGGGTAGGC
M13	AATGAAATAC to CCGTCCCGCA
M13Spf	aagCTAGAATCTCCAAATGAAATACTGGGGTAGGCCTTGTTTA
M13Spr	agcTAAACAAGGCCTACCCCAGTATTTCATTTGGAGATTCTAG
M13Donor-SOEr	TACCCCAtgcgggacggTGGAGATTCTAGTGACATAA
M13Donor-SOEf	ATCTCCAccgtcccgcaTGGGGTAGGCCTTGTTTATG
M14	TGGGGT to GTTTTG
M14Spf	The same as M13Spf
M14Spr	The same as M13Spr
M14Donor-SOEr	ACAAGGCCTcaaaacGTATTTCATTTGGAGATTCTAG
M14Donor-SOEf	ATGAAATACgttttgAGGCCTTGTTTATGACACAACT
P1	GAATTCCATATGATAGTGATGCGAGTTAAGGTAAAC
P2	GGGGATCTCGAGTCATTCGAGATCATATAATATTTTCTCGTC
P3	GATCCACCCGCGAACTATGGAAATTCCTAGCAAACAGATTGACTATAGAGACC
P4	CCCCGCTCGAGTTGTGAAACTTCTATGCTACTAACTTTCATATAAATGTCG
P5	TTGTTATAATTAGATAACCATTATACAAAAGAATTGTATAAGAATGAAAT
P6	ATTTCATTCTTATACAATTCTTTTGTATAATGGTTATCTAATTATAACAA
P7	ATAATATTTGAAATTAAATGCTTAGTGAATATACGTACAGAATAAAAATT
P8	AATTTTTATTCTGTACGTATATTCACTAAGCATTTAATTTCAAATATTAT
P9	TTGTTATAATTCTCGCCAACGTATACAAAAGAATTGTATAAGAATGAAAT
P10	ATTTCATTCTTATACAATTCTTTTGTATACGTTGGCGAGAATTATAACAA
P11	TTGTTATAATTAGATAACCATGCGCACCCCTAATTGTATAAGAATGAAAT

P12	ATTTCATTCTTATACAATTAGGGGTGCGCATGGTTATCTAATTATAACAA
P13	TTGTTATAATTCTCGCCAACGGCGCACCCCTAATTGTATAAGAATGAAAT
P14	ATTTCATTCTTATACAATTAGGGGTGCGCCGTTGGCGAGAATTATAACAA
P15	TTGTTATAATTAGATAACCATTATACAAAAGCCGGTGCGCCGAATGAAAT
P16	ATTTCATTCGGCGCACCGGCTTTTGTATAATGGTTATCTAATTATAACAA
P17	AACCATTATACAAAAGAATT
P18	AATTCTTTTGTATAATGGTT
P19	ATAATTAGATAACCATTATACAAAAGAATTGTATAAGAAT
P20	CCCCCCCCCCATTCTTATACAATTCTTTTGTATAATGGTTATCTAATTATCCCCCCCCCC
P21	TATTTTGAGCAACATTTTGTTATAATTAGATAACCATTATACAAAAGAATTGTATAAGAATGAAATCAAAGCTTATTTTATGTCACTAGAATCTCCAAATGAAATACTGGGGTAGGCCTTGTTTATGACACAACTTACAT
P22	ATGTAAGTTGTGTCATAAACAAGGCCTACCCCAGTATTTCATTTGGAGATTCTAGTGACATAAAATAAGCTTTGATTTCATTCTTATACAATTCTTTTGTATAATGGTTATCTAATTATAACAAAATGTTGCTCAAAATA
P23	AATTCCTGCAGCCCGGGGGATCCTCAGCGACACCCCTTCGGTTCCATTGGAGATTATTT TGAGCAACATTTTGTTATAATTAGATAACCATTATACAAAAGAATTGTATAAGAATGAAA TCAAAGCTTATTTTATGTCACTAGAATCTCCAAATGAAATACTGGGGTAGGCCTTGGCGGCC
P24	GGCCGCCAAGGCCTACCCCAGTATTTCATTTGGAGATTCTAGTGACATAAAATAAGCTTTGATTTCATTCTTATACAATTCTTTTGTATAATGGTTATCTAATTATAACAAAATGTTGCTCAAAATAATCTCCAATGGAACCGAAGGGGTGTCGCTGAGGATCCCCCGGGCTGCAGGAATT
P25	TTGTTATAATTAGATAACCATTATACAAAAGAATTGTATA
P26	ATTTCATTCTTATACAATTCTTTTGTATAATGGTTATCTA 3’
P27	TTGTTATAATTAGATAACCATTATACAAAA
P28	ATTTCATTCTTATACAATTCTTTTGTATAA
P29	TTGTTATAATTAGATAACCA
P30	ATTTCATTCTTATACAATTC
P31	GTTAAGGTAAACGAAAAACAAGCCGACATGATTATTGATAAACTT
P32	ACTATAATAAAAGAAGACAATGAAGCCATAGTAGACGAGAAAATATTATAT
P33	AAATGGATATATCTTGGCAAAACCGCGCCTATACAGAATATGCCAAATCCT
P34	GGAAAATATTGGTACAAATTAGAGGCAATTGGGGGGGCGTTAGCATGGATATATCTTGGCAAAACC

P35	CCCGGAATTCATGAGTGATATAATTGATGAAGTTATTTCTTCATTTAGAACTTC
P36	ACGCGTCGACTCAACCCCAGAGATCGGCAAACCTACTATCGCTTTCTATTAAAG
P37	CCCGGAATTCATGGAAATTCCTAGCAAACAGATTGACTATAGAGACCTC
P38	ACGCGTCGACTTAGACTTTTTTGTAACATTCTGGTTTTGCTTCG
P39	CCCGGAATTCATGATAGTGATGCGAGTTAAGGTAAACGAAAAACAATTCG
P40	CCGCGGATCCTCATTCGAGATCATATAATATTTTCTCGTCTACTATGTATTC
P41	CCCGGAATTCATGGAAATTCCTAGCAAACAGATTGACTATAGAGACCTC
P42	ACGCGTCGACTTATTGTGAAACTTCTATGCTACTAACTTTCATATAAATGTCG
P43	CCCGGAATTCATGAAAGTACTAGATGAAGTAACAATCTCTGAAGAAGATG
P44	ACGCGTCGACTTAATCTATATCTATTTTTCCACTTTCCATGTCAACTCCTACG
P45	CCCGGAATTCATGACGATAATGACTGGAAAACCTAAAAGTGCTAGAGAGAAAATG
P46	CCGCGGATCCTTAGACTTTTTTGTAACATTCTGGTTTTGCTTCGTATATTATACC
P47	ACGCGTCGACTTAAGGTATTCCTACAATTCTAACGTGAACCTTTTCTATATC
P48	CCCGGAATTCATGAGAGTTATAGAACTTAGAAAAATAAGAAGTACTGATATAGA
P49	A-(i5Br-dU)-TA-(i5Br-dU)-ACAAAAG
P50	C-(i5Br-dU)-TTTGTA-(i5Br-dU)-AAT
UCM30TOP	GCTTGGTTTA<u>**ATTATACAAAC**</u>TCAATATTT
UCM30BOTbiotin [	[Btn]AAATATTGAGTTTGTATAATTAAACCAAGC
CTRLTOP	GCTTGGTTTA<u>**CGGCGCACCCA**</u>TCAATATTT
CTRLBOT	[Btn]AAATATTGATGGGTGCGCCGTAAACCAAGC
