# Supplementary material for: An archaeal nucleoid-associated protein binds an essential motif in DNA replication origins
Source: Nat Commun. 2025 Jun 5;16:5230. doi: 10.1038/s41467-025-60618-3 (PMC12141716; doi:10.1038/s41467-025-60618-3)
Supplement: Supplementary file 2 — Description of Additional Supplementary Files [file 41467_2025_60618_MOESM2_ESM.pdf]

Description of Additional supplementary file

Supplementary Data 1.

Pairwise comparisons of transcriptome changes upon UBP over-expression DESeq2 analysis of differential gene expression in the various pairwise combinations of empty-vector containing (Vec) and UBP-overexpressing strains (UBP) at 30, 45 and 55 hours of growth in inducer-containing medium.
